# The Great Smog Month and Spatial and Monthly Variation in Air Quality in Ambient Air in Delhi, India

**DOI:** 10.5696/2156-9614-10.27.200910

**Published:** 2020-08-25

**Authors:** Anchal Garg, N.C. Gupta

**Affiliations:** University School of Environment Management, Guru Gobind Singh Indraprastha University, New Delhi, India

**Keywords:** spatial-temporal variations, ambient air pollution, AQI, stubble burning, particulate matter, NO_2_, episodic event

## Abstract

**Background.:**

In recent years, poor urban air quality in Delhi, India has gained significant attention. Episodic events including crop stubble burning and Diwali celebrations are considered major factors in the worsening quality of ambient air.

**Objective.:**

This study aimed to investigate spatial and monthly variation as well as the role of episodic events in ambient air quality in Delhi, including the ‘Great Smog' month of November 2017.

**Methods.:**

Monitoring of air pollutants (particulate matter (PM_10_, PM_2.5_, PM_1_) and nitrogen dioxide (NO_2_)) was carried out at three distinct locations of Delhi from April 2017–February 2018. The concentration of NO_2_ was measured using a modified Jacob and Hochheiser method and PM was measured using a GRIMM aerosol spectrometer. Air quality index was also determined to identify the effects of air pollution on human health.

**Results.:**

Overall, the levels of air pollution were found to be approximately 2.1–3.2 times higher along a traffic intersection and about 1.4–2.0 times higher in a commercial area compared with an institutional area. The highest average monthly concentrations of PM_10_, PM_2.5_, PM_1_ and NO_2_ were 768, 374, 298 and 149 μg/m_3_, respectively, during the Great Smog month of November 2017. November and August were recorded as the most polluted and cleanest months, respectively, in the city. Generally, poor to severe categories of the air quality index (AQI) were obtained from October to February. Higher concentrations during November were attributed to stubble burning in the nearby states of Delhi with the additive effect of fireworks during Diwali celebrations.

**Conclusions.:**

Severe ambient air quality as observed in the present study is a serious matter of concern for the health of Delhi's population. To control spikes in poor air quality during episodic events, it is imperative to raise awareness among farmers regarding the severe health hazards of stubble burning.

**Competing Interests.:**

The authors declare no competing financial interests.

## Introduction

It is estimated that every year around 4.2 million deaths are attributed to ambient air pollution worldwide, as approximately 91% of the world's population lives in places where air quality levels exceed World Health Organization (WHO) standards.^1^ Urbanization, industrialization, growth in population, increases in the number of automobiles and construction activities are the leading causes for increased air pollution in Asia's largest metropolitan areas. According to various studies, the air quality in Delhi, the capital city of India, was found to be the worst of any major city in the world.^2^–^5^ Particulate matter (PM) of aerodynamic diameter less than or equal to 10 μm (PM_10_) and 2.5 μm (PM_2.5_) are considered major pollutants responsible for deteriorating urban air quality. Various studies have correlated the adverse effects of exposure to finer particles, in the range of PM_1_ to PM_2.5_, on the respiratory and cardiovascular system. These finer particles have various long-term and short-term effects on human health including asthma, coughing, sneezing, heart attack, cancer, etc., whereas exposure to coarse particles (PM_10_) has been linked with an increase in respiratory disease.^6^–^11^ As per the Indian National Ambient Air Quality Monitoring Program (NAAQM), after PM, nitrogen dioxide (NO_2_) has the second highest exceedance rate in India.^12^ Exposure to NO_2_ may lead to various respiratory problems such as wheezing, coughing, colds, flu and bronchitis.^13^

Air pollutant levels fluctuate by time of year and location. Pollutants in ambient air exhibit significant spatial and seasonal variations due to differences in human activities, topography, weather conditions and land cover.^14^–^17^ In addition to spatial-temporal variations, there are also episodic events which generally cause two to four day spikes during which the concentration of pollutants in ambient air reaches alarming levels.

In India, these events include the celebration of Diwali, storm events and open crop stubble burning.^18^ In November 2017, there was an event known as the “Great Smog of Delhi”, when the levels of PM_2.5_ and PM_10_ peaked to 999 μg/m^3^, compared to the safe limits for these pollutants of 60 and 100 μg/m^3^, respectively.^19^ Increased crop stubble burning was considered to be most responsible for this episodic event. Despite an official ban, the practice of stubble burning is very common in the northern region of India. It is estimated that every year, around 35 million tons of crop residues are burnt in Punjab and Haryana. The smoke produced during this period greatly contributes to Delhi's smog; and is worse during cold climatic conditions as cool temperatures trap pollution. Every year the air quality of Delhi worsens due to smog.^18^,^20^,^21^

The present study used the air quality index (AQI) as a tool to measure the quality of air in Delhi. It focuses on the possible effects on human health after exposure to unhealthy air within a few hours or days.^22^ High levels of air pollutants indicate a high AQI value which corresponds to greater human health effects.^23^ There are six different classified AQI categories,: good (0–50, minimal impacts), satisfactory (51–100, minor breathing discomfort to sensitive people), moderately polluted (101–200, breathing discomfort to people with heart and lung disease such as asthma), poor (201–300, breathing discomfort to most people from prolonged exposure), very poor (301–400, causes respiratory illness from prolonged exposure) and severe (401–500, affects healthy people and seriously impacts those with preexisting diseases).^22^–^23^ The color codes defined for good, satisfactory, moderately polluted, poor, very poor and severe air quality are dark green, light green, yellow, dark yellow, red and dark red, respectively.^24^ The present study was designed to assess spatial and monthly variation in air pollution as well as the effect of smog from stubble burning on pollution levels in the ambient air of Delhi. In addition, the AQI was estimated for identifying air quality and its impacts on human health.

**Abbreviations**
ANOVA: Analysis of variance
AQI: Air quality index
CPCB: Central Pollution Control Board
NAAQS: National Ambient Air Quality Standards
PM: Particulate matter

## Methods

**Figure 1:**
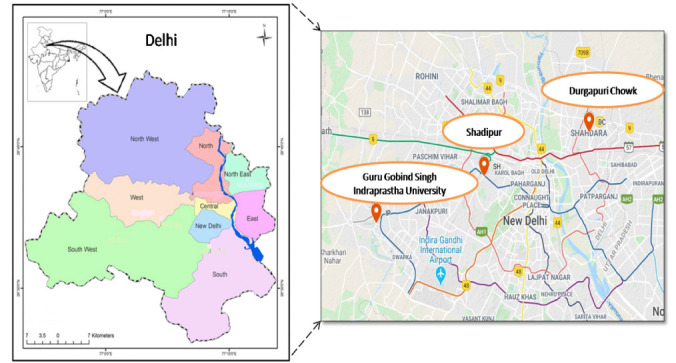
Representation of sampling locations in Delhi

Delhi, the capital city of India, situated at 28.7041°N and 77.1025°E and spread over an area of 1484 km^2^, was selected for the present study. It is situated at an altitude of 293 m above mean sea level and has a sub-tropical climate consisting of four well-defined seasons: summer (April–June), monsoon (July–September), autumn (October–November) and winter (December–February). There is a large temporal variation in the climate of the city. The summer season experiences windy conditions and temperature reaches as high as 48°C, whereas winter is characterized by calm conditions and temperature as low as 3–4°C. Due to southwestern monsoon winds, Delhi receives most of its rainfall (average annual rainfall of 714 mm) during July to September.^25^ To understand the spatial-temporal variation and episodic rise of air pollution in the study region, monitoring was carried out at three distinct locations: Durgapuri Chowk (traffic congestion area), Shadipur (commercial area) and Guru Gobind Singh Indraprastha University (institutional area). These locations are shown in Figure 1.

### Measurement of pollutants

Monitoring of PM_10_, PM_2.5_, PM_1_ and NO_2_ was carried out from April 2017–February 2018. Every month two samples were collected from each location; 22 samples from a single location and a total of 66 samples during the study period. Monitoring was conducted between 9:00–13:00 hours. The concentration of NO_2_ was measured using a respirable dust sampler (Envirotech APM 460 BL) with a flow rate of 1 lmin^−1^. A modified Jacob and Hochheiser method was used for measurement and analysis of NO_2_.^26^,^27^ Levels of PM_10_, PM_2.5_ and PM_1_ were measured with an aerosol spectrometer (GRIMM, Model 1.108, Germany). The working principle of this instrument involves orthogonal scattering of light.^28^

### Meteorological data

Meteorological parameters like wind speed, wind direction, relative humidity and ambient temperature were obtained from the Continuous Ambient Air Quality Monitoring Station of the Central Pollution Control Board (CPCB).

Daily measurements from these meteorological parameters were converted into monthly average values and wind rose diagrams were plotted using wind speed and direction data.

### Calculation of AQI

In the present study, the AQI was determined by considering the concentration of three criteria pollutants: PM_10_, PM_2.5_ and NO_2_. The AQI calculation method involved formation of sub-indices for each pollutant and aggregation of sub-indices using Equation 1.^29^

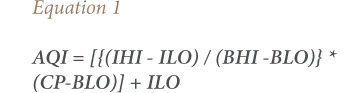
where, AQI is the air quality index for pollutant “P”; CP is the actual ambient concentration of pollutant “P”; BHI is the breakpoint concentration greater or equal to given concentration; BLO is the breakpoint concentration smaller or equal to given concentration; IHI is the AQI value corresponding to BHI; and ILO is the AQI value corresponding to BLO.

### Statistical interpretation

To validate the spatial and monthly variation in air pollutants, statistical analysis was carried out using Microsoft Excel and the Statistical Package for the Social Sciences (SPSS) software. Descriptive statistics, two-way analysis of variance (ANOVA), Pearson's correlation analysis, regression analysis, and t-statistics were applied in the present analysis.

## Results

**Figure 2:**
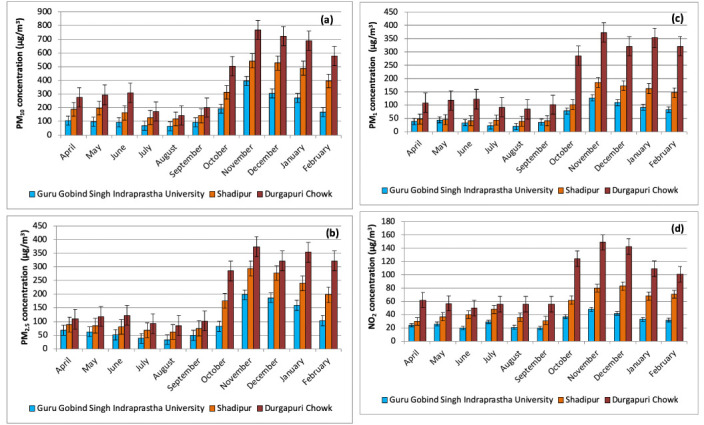
Spatial-temporal variations across different pollutants (a) PM_10_, (b) PM_2.5_, (c) PM_1_ and (d) NO_2_

The average levels of PM_10_, PM_2.5_, PM_1_ and NO_2_ along with their descriptive statistics from April 2017–February 2018 at all three locations are shown in Table 1. The spatial-temporal variations of these pollutants are presented in Figure 2. Significant spatial variations were observed for all pollutants among three study locations for each season. In addition, as seen in Table 1, for all the air pollutants, the mean values were higher than the median value, making the distribution positively skewed. The concentration distribution was asymmetric and relates to the large seasonal fluctuations in concentrations. Overall, the level of air pollution was found to be 2.1–3.2 times higher in Durgapuri Chowk, a heavy traffic intersection area and 1.4–2.0 times higher in Shadipur, a commercial area, compared to Guru Gobind Singh Indraprastha University, an institutional area.

**Table 1:** Descriptive Statistics of Air Quality Parameters

**Month**	**PM_10_ (μg/m^3^)**	**PM_2.5_ (μg/m^3^)**	**PM_1_ (μg/m^3^)**	**NO_2_ (μg/m^3^)**	AQI^*^
April	190.33	88.33	51.33	38.67	194
May	196.33	87.90	53.00	40.00	188
June	188.00	84.33	49.67	36.67	179
July	122.67	65.67	42.67	44.33	133
August	109.00	59.67	40.00	37.67	120
September	145.67	75.33	45.67	35.67	161
October	336.33	181.33	126.00	74.33	336
November	568.67	289.00	204.00	92.33	574
December	518.67	262.33	162.67	89.33	547
January	483.33	251.33	144.67	70.00	509
February	380.33	207.67	131.00	68.00	396
Mean	294.33	150.33	95.33	57.00	304
Median	203.67	92.67	54.67	46.33	194
SD	171.33	90.67	60.33	22.33	176
Kurtosis	−1.08	−1.46	−1.07	−1.22	−1.52
Skewness	0.64	0.59	0.64	0.52	0.57
Minimum	109.00	59.67	39.67	33.33	120
Maximum	568.67	289.00	204.00	93.33	574

^*^The color codes indicating good, satisfactory, moderately polluted, poor, very poor and severely polluted air quality are dark green, light green, yellow, dark yellow, red and dark red, respectively.^24^

**Figure 3:**
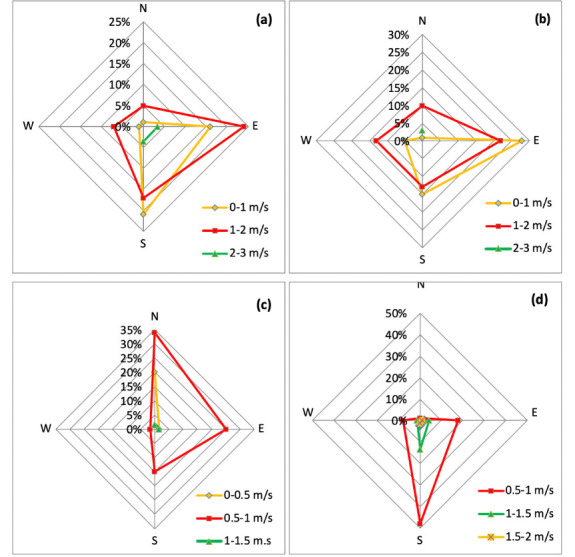
Wind-rose diagrams in different seasons (a) summer, (b) monsoon, (c) autumn and (d) winter

The wind rose diagrams *(Figure 3)* indicate that during autumn 55% of the time the wind blows from the north, while in winter, 65% of the time the wind blows from the south. During the summer and monsoon months, the direction of the wind shifts from south to southeast. The majority of the time, the wind speed was less than 1 m/s during autumn and winter, and greater than 1 m/s during the summer and monsoon seasons.

**Figure 4:**
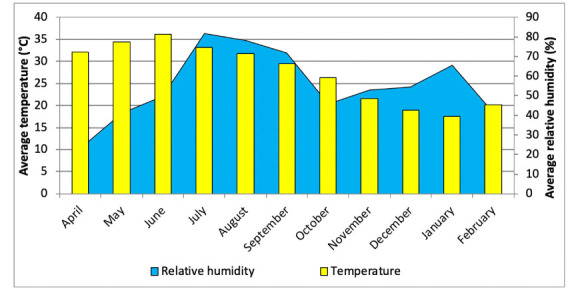
Monthly variation in ambient temperature and relative humidity

Other meteorological parameters like relative humidity and ambient temperature also exhibited significant monthly variations. During the study period, monthly average ambient temperature and relative humidity varied from 17.6–36.2ºC and 23.1–81.7%, respectively *(Figure 4).* Minimum and maximum values of relative humidity occurred in April and July while minimum temperature was recorded in January and maximum in June.

### Episodic levels of air pollutants during November-“The Great Smog month” in Delhi

**Figure 5:**
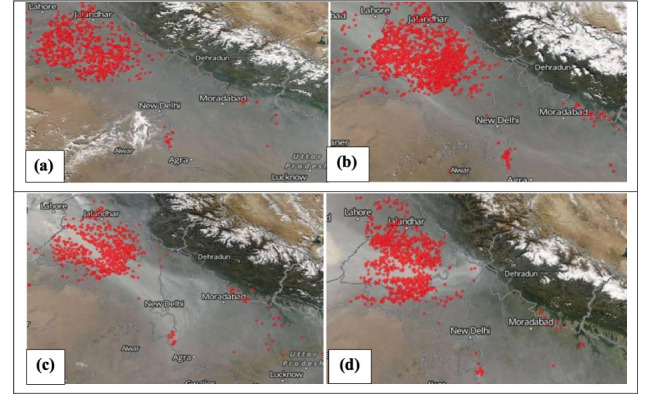
Satellite imagery during crop stubble burning (red dots) in Punjab (a) 27 October, (b) 29 October, (c) 31 October, (d) 5 November, 2017

**Figure 6:**
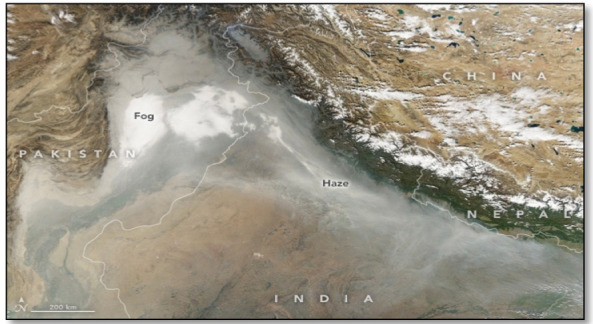
Haze and fog blanketing northern India

The concentration of air pollutants in Delhi spikes during episodic events including the celebration of Diwali and crop burning in other states, which results in rapid increases in pollution levels. Maximum average monthly concentrations of PM_10_, PM_2.5_, PM_1_ and NO_2_ (768, 374, 298 and 149 μg/m^3^, respectively) were recorded in the month of November in Durgapuri Chowk. During November PM_10_, PM_2.5_, and NO_2_ levels were recorded approximately 5–14, 5–7 and 2 times higher than the 24-hour National Ambient Air Quality Standards (NAAQS) proposed by the CPCB of India, respectively.^19^ Data obtained through satellite imagery released by the National Aeronautics and Space Administration (NASA) clearly show that crop burning was mainly concentrated in Punjab and intensified in the latter half of October *(Figure 5 (a),(b) and (c)).*^30^ The satellite images very clearly establish the relationship between the drastic rise in pollution level during this month with stubble burning in the agrarian belts of Punjab and Haryana. In addition, during November 2017, due to crop stubble burning, smoke was spreading across northern India, and as decreased wind speed and cooling weather mixed the smoke with fog and dust it formed a thick haze *(Figure 6).*

### Air quality index at different locations

**Figure 7:**
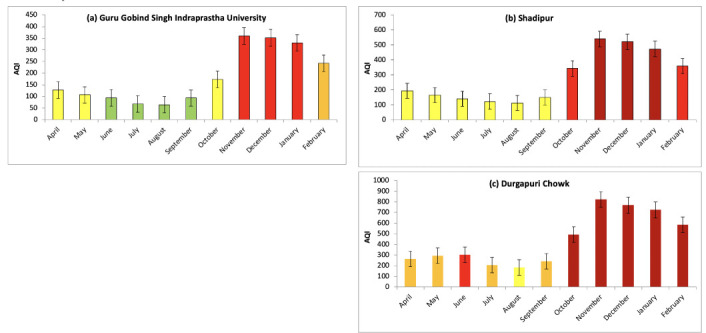
Temporal variations in air quality index (a) Guru Gobind Singh Indraprastha University, (b) Shadipur and (c) Durgapuri Chowk

The average AQI levels are reported in Table 1 and their variations are represented by bar diagrams in Figure 7. During the study period, AQI was observed to be lower in Guru Gobind Singh Indraprastha University with moderate quality from June–September and poor quality in April and May. However, air quality at the same location was in the severe category during November, December and January. The AQI in Durgapuri Chowk was consistently very high and in the very poor to severe category. In addition, the AQI in the Shadipur location from October to February fell under the severe category. In the present study, PM_10_ and PM_2.5_ were the most prominent air pollutants contributing to the deterioration of ambient air quality. The poor air quality in Delhi is a serious matter of concern for public health. In the present study, the AQI during the month of November ranged between 360–823 at different locations. The high AQI during this episodic period indicates worsening air quality and was associated with an increase in hospital admissions.^22^

### Statistical interpretation

The annual NAAQS prescribed by the CPCB of India are 60, 40, and 40 μg/m^3^ for PM_10_, PM_2.5_, and NO_2_, respectively, whereas the annual air quality standards prescribed by the WHO are 20, 10, and 40 μg/m^3^ for PM_10_, PM_2.5_, and NO_2_, respectively. In order to identify variations in spatial and seasonal distribution of air pollutants a statistical interpretation was conducted. In order to justify the spatial and monthly variations, a two-way ANOVA test was applied on the air pollutant data sets. The results obtained for this analysis are shown in Table 2. From Table 2, it is clear that the F-values obtained in this study are much higher compared to the F-critical level for both locations and months. These results clearly show that there is a high spatial-temporal variation in ambient air pollutants at a 0.05 level of significance.

**Table 2:** Two-way ANOVA Analysis for Ambient Air Quality Showing Spatial and Monthly Variation

**Source of variation**	**SS**	**df**	**MS**	**F**	**P-value**	**F-critical**
**PM_10_**						

Within months	864334.2	10	86433.42	18.28	6.00E-08	2.35
Within locations	356798.2	2	178399.12	37.72	1.63E-07	3.49
**PM_2.5_**						

Within months	237838.3	10	23783.83	17.85	7.34E-08	2.35
Within locations	71718.06	2	35859.03	26.92	2.13E-06	3.49
**PM_1_**						
Within months	105312.2	10	10531.22	15.43	2.55E-07	2.35
Within locations	25406.61	2	12703.30	18.62	2.72E-05	3.49
**NO_2_**						

Within months	14736.67	10	1473.67	5.97	3.50E-04	2.35
Within locations	18327.45	2	9163.73	37.10	1.86E-07	3.49
**AQI**						

Within months	925097.6	10	92509.76	21.47	1.47E-08	2.35
Within locations	380486.2	2	190243.12	44.18	4.62E-08	3.49

Abbreviations: df, degrees of freedom; MS, mean square; SS, sum of square

Similarly, one sample t-test (95% CI) was applied for identifying the variation in the level of PM_10_, PM_2.5_ and NO_2_ from 24-hour and annual NAAQS *(Table 3)* as prescribed by the CPCB of India. It was found that the obtained t-statistics values were higher than t-critical for PM_10_ and PM_2.5_ for both 24-hour and annual NAAQS suggesting that these values were always much higher than the NAAAQS. However, for NO_2_ the obtained t-value (−3.83) was less than the 24-hour NAAQS value which shows that on an average basis, NO_2_ levels were found to be within the permissible limit (80 μg/m^3^).

**Table 3:** One Sample T-Test Showing Variation in Mean Level of Pollutants from 24-Hour and Annual National Ambient Air Quality Standards

**Values**	**24 hours NAAQS value**			**Annual NAAQS value**		

	**PM_10_**	**PM_2.5_**	**NO_2_**	**PM_10_**	**PM_2.5_**	**NO_2_**
Mean (μg/m^3^)	294.48	150.30	57.00	294.48	150.30	57.00
Variance (μg/m^3^)	41116.38	10506.22	1187.63	41116.38	10506.22	1187.63
Observations	33.00	33.00	33.00	33.00	33.00	33.00
NAAQS value (μg/m^3^)	100.00	60.00	80.00	60.00	40.00	40.00
df	32.00	32.00	32.00	32.00	32.00	32.00
t-statistics	5.51	5.06	−3.83	6.64	6.18	2.83
P(T<=t) one-tail	2.25E-06	8.32E-06	2.79E-04	8.55E-08	3.21E-07	3.95E-03
t critical one-tail	1.69	1.69	1.69	1.69	1.69	1.69
P(T<=t) two-tail	4.51E-06	1.66E-05	5.57E-04	1.71E-07	6.42E-07	7.90E-03
t-critical two-tail	2.04	2.04	2.04	2.04	2.04	2.04

Abbreviation: NAAQS= National Ambient Air Quality Standards

**Figure 8:**
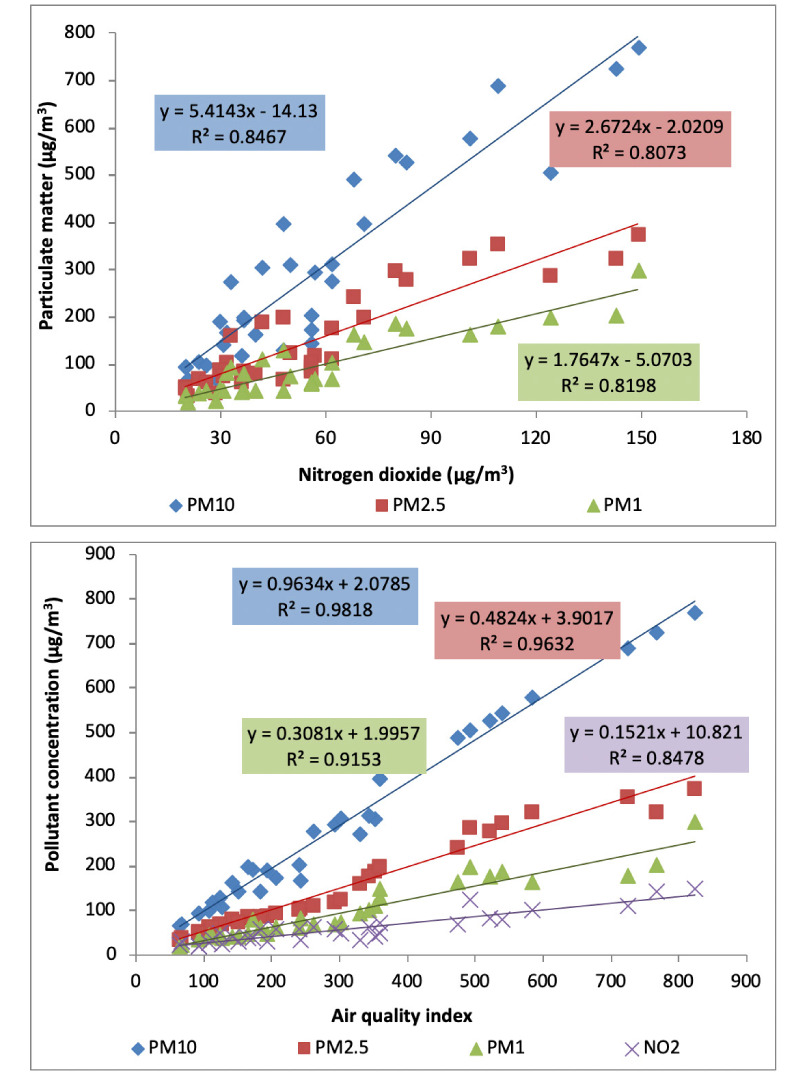
Scatter plots showing regression analysis between pollutants and AQI

The scatter plots *(Figure 8)* show the best fitting line between various pollutants and AQI.

The results of correlation analysis *(Table 4)* showed a strong positive R^2^ value between ambient air pollutants, indicating common emission sources of these pollutants, particularly vehicular emissions.

**Table 4:** Pearson Correlation Analysis for Air Pollutants and AQI

	PM_10_	PM_2.5_	PM_1_	NO_2_	AQI
PM_10_	1.000				
PM_2.5_	0.984	1.000			
PM_1_	0.958	0.965	1.000		
NO_2_	0.920	0.898	0.905	1.000	
AQI	0.991	0.981	0.957	0.921	1.000

## Discussion

The reason for spatial variations was the difference in the population size, the pattern of land use, vehicular and traffic density and presence of small-scale industries.^31^ Through spatial distribution analysis, we identified the highest concentration of air pollutants and AQI at Durgapuri Chowk, which may be attributed to the high traffic density, vehicular pattern, traffic congestion and large population. Durgapuri Chowk is in northeast Delhi and as reported in a study conducted on ambient air in Delhi, east and northeast Delhi are home to nearly a quarter of the total population with a density of 36000 person per km^2^.^32^,^33^ Guru Gobind Singh Indraprastha University, on the other hand, is a newly settled area of southwest Delhi. The area near Guru Gobind Singh Indraprastha University has a smaller population density and is covered with more green spaces. Shadipur is a commercial location with various construction activities in the area, resulting in an accumulation of particles in ambient air.

Previous studies revealed that seasonal variations are greatly influenced by meteorological conditions like relative humidity, ambient temperature, wind speed, and wind direction.^31^–^34^ In terms of seasonal distribution, the celebrations of Diwali and stubble burning during the autumn season were the main cause for spikes in PM concentrations.^18^,^38^ Other than November, the highest pollutant concentrations occurred during the winter months and a general trend of lower values occurred during the monsoon season. The reason for such high concentrations during autumn and winter was the calm and stable atmospheric conditions with lower mixing height, lower wind speed and decreased ambient temperature, hence, air pollutants were not able to disperse into the atmosphere and led to higher concentration of pollutants in ambient air.^34^,^39^–^41^ During the monsoon season, frequent rain washes down the airborne particulates and results in a reduction in PM concentrations.

Concentrations of air pollutants during November were recorded to be much higher and essentially a veritable gas chamber hovered over Delhi. Thus, November 2017 was considered the “The Great Smog month of Delhi” with the highest air pollution levels in history in India. The sources for such high concentrations during the month of November were not only heavy construction activities and vehicular pollution but also stubble burning in nearby states.^18^ The inversion conditions in the atmosphere during November prevents pollution from dispersing into the atmosphere and result in worsening air quality. According to WHO guidelines, exposure to such high concentrations is very dangerous to the health of the elderly and children, hence the Government of Delhi decided to shut down 6,000 schools during the worst days.^42^ In order to control such spikes, collaboration with neighboring states is essential for effective improvement of Delhi's air quality and public health.^43^

### Study limitations

Limitations of the present study include the small number of sites (three) and the small sample size.

## Conclusions

Delhi's atmosphere is becoming severely polluted due to increased urbanization, vehicles, industries, construction and burning activities within the territory region and adjoining areas. The present study identified levels of air pollutants and analyzed spatial-temporal variations and found that the air quality in Delhi is worsening with time. Two-way ANOVA testing showed significant spatial and monthly variations in different air pollutants. One sample t-test results showed that pollutants exceeded the standard values prescribed by the pollution control agency of India. The results of the present analysis show that PM_10_ and PM_2.5_ are the most prominent pollutants in ambient air in Delhi. During the summer and with windy conditions, these pollutants are barely maintained within the standard prescribed by NAAQS. The positive correlation obtained between air pollutants and AQI showed similar emission sources, particularly vehicles. The poor to severe range of AQI obtained during November is a serious matter of concern for the health of Delhi's residents. Spatial variations reveal that conditions near roads are worse due to more congested traffic density and vehicular mobility. The spike in pollution levels during the winter season and episodic events are extremely concerning. To control spikes during stubble burning, it is necessary to raise awareness among farmers regarding the health hazards of stubble burning and new methods of farming waste disposal should be explored which may additionally allow farmers to earn extra money by selling material made from the waste. Government and non-governmental organizations should become involved in active planning with proper coordination and execution within the farming sector and in major policy intervention sectors. Management strategies include alternative fuel types, improvements in fuel quality, and implementation of odd-even schemes, where private vehicles with registration numbers ending with an odd digit are allowed on roads on odd dates and those with an even digit on even dates. Controlling unnecessary construction activities, managing vehicular patterns, proper traffic management and availability of better public transportation linked with road networks are necessary steps to combat these major urban environmental hazards.
